# *Drosophila* NMNAT Maintains Neural Integrity Independent of Its NAD Synthesis Activity

**DOI:** 10.1371/journal.pbio.0040416

**Published:** 2006-11-28

**Authors:** R. Grace Zhai, Yu Cao, P. Robin Hiesinger, Yi Zhou, Sunil Q Mehta, Karen L Schulze, Patrik Verstreken, Hugo J Bellen

**Affiliations:** 1 Howard Hughes Medical Institute, Baylor College of Medicine, Houston, Texas, United States of America; 2 Department of Molecular and Human Genetics, Baylor College of Medicine, Houston, Texas, United States of America; 3 Program in Developmental Biology, Baylor College of Medicine, Houston, Texas, United States of America; Austrian Academy of Sciences, Austria

## Abstract

Wallerian degeneration refers to a loss of the distal part of an axon after nerve injury. *Wallerian degeneration slow (Wld^s^)* mice overexpress a chimeric protein containing the NAD synthase NMNAT (nicotinamide mononucleotide adenylyltransferase 1) and exhibit a delay in axonal degeneration. Currently, conflicting evidence raises questions as to whether NMNAT is the protecting factor and whether its enzymatic activity is required for such a possible function. Importantly, the link between *nmnat* and axon degeneration is at present solely based on overexpression studies of enzymatically active protein. Here we use the visual system of *Drosophila* as a model system to address these issues. We have isolated the first *nmnat* mutations in a multicellular organism in a forward genetic screen for synapse malfunction in *Drosophila*. Loss of *nmnat* causes a rapid and severe neurodegeneration that can be attenuated by blocking neuronal activity. Furthermore, in vivo neuronal expression of mutated *nmnat* shows that enzymatically inactive NMNAT protein retains strong neuroprotective effects and rescues the degeneration phenotype caused by loss of *nmnat*. Our data indicate an NAD-independent requirement of NMNAT for maintaining neuronal integrity that can be exploited to protect neurons from neuronal activity-induced degeneration by overexpression of the protein.

## Abstract 2

The first mutant analysis of NMNAT (nicotinamide mononucleotide adenylyltransferase 1) reveals an essential neuronal protective role that functions independently of NMNAT's enzymatic activity. NMNAT can also be exploited to protect neurons against activity-induced neurodegeneration.

## Introduction

Following nerve injury, distal axons and synaptic terminals undergo Wallerian degeneration within 48 h [1]. This process occurs in both the central and peripheral nervous systems and has several key features: (1) the distal stump of an injured axon loses its ability to transmit action potentials; (2) the axon and synaptic terminal become fragmented; (3) the cytoskeleton undergoes granular degeneration; and (4) the axonal and nerve terminal debris are removed by Schwann cells and invading macrophages [2]. Recent studies have started to unveil the molecular mechanisms of Wallerian degeneration. Inhibiting ubiquitination delays axon degeneration, suggesting the involvement of ubiquitin-mediated proteasomal degradation in the early stages of Wallerian degeneration [3]. In addition, studies have suggested that Wallerian degeneration is distinct from the apoptosis pathway, because caspases are not involved in Wallerian degeneration, and overexpression of apoptosis-inhibiting factors and mutations that block apoptosis fail to prevent axonal degeneration [4–6].

The discovery of the slow Wallerian degeneration mutant *(Wld^s^)* mouse, in which Wallerian degeneration is delayed by 2–3 wk, has provided a genetic inroad to study the mechanisms of deterioration and protection in axonal degeneration [7–10]. The pronounced delay in degeneration is caused by a tandem triplication of an 85-kilobase (kb) region, resulting in the overexpression of a chimeric *Ube4b/Nmnat* gene, which contains the amino terminal 70–amino acid fragment of *Ube4b* (ubiquitination factor E4B), the entire coding sequence of *Nmnat1,* and a unique 18–amino acid linking region translated from the 5′ untranslated region (UTR) of *Nmnat1* [11,12]. Overexpression of the fusion protein in transgenic mice and rats reproduces the Wld^S^ phenotype [12,13], and recent studies show that overexpression of this fusion protein in *Drosophila* also protects axons from degeneration [14]. However, there are conflicting reports as to whether Nmnat1 alone is the protective factor [15–18], because transgenic mice overexpressing *Nmnat1* do not exhibit protection from Wallerian degeneration [15,16]. In contrast, a detailed analysis of Wld^s^ chimeric protein in the central nervous system (CNS) suggests that the increased level of Nmnat1 is significant in *Wld^s^* mice and that there is no obvious change in ubiquitination. Hence, its protective effects seem to be unrelated to the ubiquitination function of Ube4b [17]. In *Drosophila,* overexpression of *nmnat* can delay axonal degeneration [18]. The role of Ube4b remains controversial, however, as transfection of N70-Ube4b in cultured neurons does not produce a protective phenotype [19]. On the other hand, a recent report indicates that N70-Ube4b binds an important proteasomal chaperone (VCP) and relocates it to the nucleus [20]. Hence, it remains to be established whether N70-Ube4b is sufficient and/or necessary for a protective phenotype.

Two in vitro studies pinpoint Nmnat1 as the protective agent and suggest that it acts either in the nucleus [19] or locally in axons [21]. Although both in vitro studies indicate a requirement of NAD for the protective effect, *Wld^s^* mice do not exhibit increased NAD levels [12]. This finding suggests either that local fluctuation of NAD levels is under the detection limit or that the Nmnat1 protein may have additional functions beyond NAD synthesis.

Understanding the normal neuronal function of endogenous Nmnat in vivo is crucial to unveiling the mechanisms of neural degeneration and neural protection offered by Wld^s^ protein. Here we show that loss of Drosophila nmnat causes severe neuronal and synaptic degeneration without affecting neural development. Importantly, this *nmnat*-dependent degeneration in photoreceptors can be attenuated by blocking phototransduction. NMNAT therefore functions to protect against activity-induced deterioration under normal conditions, and overexpression of NMNAT protects neurons from excessive activity-induced degeneration. This role is independent of its NAD synthesis activity, because overexpression of NMNAT protein with less than 1% activity can rescue the degeneration caused by loss of *nmnat* and has strong protective effects. We conclude that NMNAT is required to maintain neuronal integrity independent of its NAD synthesis function.

## Results

### Drosophila nmnat Is a Homolog of Mouse *Nmnat1* Overexpressed in *Wld^s^*

To identify genes that are involved in synapse development and function, we carried out a forward genetic screen using the *eyFLP* system [22–24]. The *eyFLP* system allows screening of flies that are homozygous for lethal mutations in the visual system but heterozygous in the rest of the animal [25]. Our primary screen was based on isolating ethyl methanesulfonate (EMS)-induced mutants that fail in a phototaxis assay, followed by screening for defects in electroretinograms (ERGs) and assessing morphological defects of photoreceptor axons and terminals [22]. We screened 210,000 flies and isolated 7,500 flies with grossly normal eye morphology that phototax poorly [26]. We next subjected the flies to ERG tests and isolated 450 mutants with abnormal ERG responses. Subsequent complementation tests yielded 64 complementation groups with two or more alleles.

**Figure 1:**
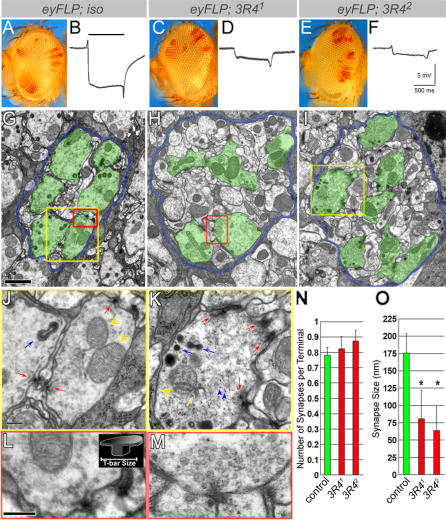
Mutations in Complementation Group *3R4* Disrupt Synaptic Structures in Photoreceptor Terminals (A–F) External morphology of the homozygous eyes of *3R4^1^* (C) and *3R4^2^* (E) are normal when compared to those of isogenized control (A). Red eye color marks heterozygous patches. (B), (D), and (F) ERG recordings of control and mutant eyes. Note the reduced depolarization and on/off response. Bar above trace in (B) indicates duration of light stimulus. (G–I) TEM micrographs of lamina cartridges containing control, *3R4^1^,* and *3R4^2^* mutant terminals, respectively. Demarcating glia are colored blue and photoreceptor terminals green to accentuate the structures. Note the photoreceptor terminals are disorganized in mutants. The yellow boxes in (G) and (I) indicate the regions shown in (J) and (K), respectively. The red boxes in (G) and (H) indicate the regions shown in (L) and (M), respectively. Scale bar in (G) for (G–I) indicates 1 μm. (J) and (K) Individual terminals that are boxed in (G) and (I) (yellow boxes). *nmnat* mutant terminals have amorphic active zone structures (red arrows), aberrant capitate projections (blue arrows), aberrant mitochondria (yellow arrowheads), and abnormal membranes (yellow arrow), as well as an aberrant cytoskeleton (blue arrowheads), which are not observed in wild-type terminals. Scale bar indicates 200 nm. (L) and (M) Individual active zones that are boxed in (G) and (H) (red boxes). Compared to the clearly defined wild-type active zone structure (L), *nmnat* mutant active zones are amorphic and reduced in size. Both wild-type and mutant T-bars are surrounded by synaptic vesicles. Scale bar indicates 200 nm. (N) and (O) Quantification of synapse number and size. No significant difference was found in the average number of active zones per terminal between control (118 terminals counted), and *3R4^1^* (93 terminals counted) or *3R4^2^* (71 terminals counted). Synapse size was measured by the width of T-bar platform profile (L) (insert). The size of T-bars in either *3R4^1^* (23 measured) or *3R4^2^* (31 measured) is significantly reduced compared to the control (38 measured). An asterisk (*) indicates *p* < 0.05.

The *Drosophila* compound eye consists of 800 unit eyes, or ommatidia, each of which contains eight photoreceptors. Photoreceptors 1–6 (R1–R6) project to the first optic neuropil, or lamina, whereas R7 and R8 project deeper into the brain [27]. To isolate genes that specifically regulate synapse assembly and maintenance, we carried out a systematic transmission electron microscopy (TEM) analysis to uncover mutants with abnormal synapse structure in R1–R6 terminals [28]. Out of 60 mutants analyzed by TEM, two homozygous lethal mutations with abnormal active zone morphology that fail to complement each other *(3R4^1^* and *3R4^2^)* were isolated. As shown in Figure 1A, 1C, and 1E, eyes that are mostly homozygous mutant (white areas) for either of the two alleles of complementation group *3R4* are smooth and have a normal external morphology, suggesting that eye development is normal. ERGs, extracellular recordings that measure the response of photoreceptor neurons to a light stimulus, were performed on animals with eyes mutant for either allele. *3R4* mutants exhibit a reduced depolarization and reduced on/off response in young animals (Figure 1B, 1D, and 1F), suggesting that the mutant photoreceptors have an impaired ability to respond to light and to elicit a postsynaptic response. We next examined the ultrastructure of lamina synapses where the ERG response is generated. As shown in Figure 1G–1I, TEM of lamina synapses of 1-d-old flies reveals morphological defects. In contrast to the organized cartridge structure in control lamina with six photoreceptor terminals per cartridge (Figure 1G), the mutant laminae are disorganized, with various number of terminals (Figure 1H and 1I). Within the terminals, we observe a fragmented cytoskeleton and misshapen membrane organelles (Figure 1K). More interestingly, the mutant synaptic structure exhibits distinct phenotypes. In *Drosophila* presynaptic terminals, active zones have synaptic ribbon structures called T-bars that each consist of a “platform” and a “pedestal” [29]. In *3R4* mutants, T-bars are amorphous and less electron dense compared to wild type, but they are surrounded by clusters of normally sized synaptic vesicles (Figure 1K and 1M). Although the number of synapses per terminal is not altered, the size of the T-bar profile, measured by the width of platforms (Figure 1L, insert), is significantly reduced compared to the control (Figure 1N and 1O).

**Figure 2:**
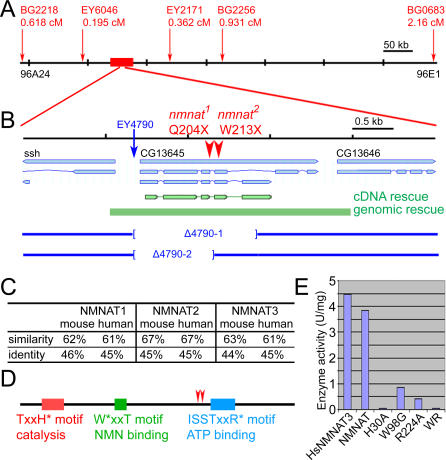
Complementation Group *3R4* Encodes *nmnat,* an Adenylyltransferase (A) Fine mapping using the *P* element recombination method. The insertion sites of the five *P* elements used to map the gene are marked by red arrows. The recombination ratio for each *P* element is listed in centiMorgans (cM). The red box marks the region delineated by fine mapping. (B) The genomic region of CG13645. The point mutations uncovered by sequencing are marked by red arrowheads. The genomic rescue construct and the cDNA rescue construct are both marked in green. The blue arrow indicates the insertion site of *P* element EY4790 used to generate the excision lines *Δ4790–1* and *-2*. The boundaries of the excised regions are marked by brackets in the blue lines. (C) *Drosophila* NMNAT protein is homologous to mouse and human NMNAT1, −2, and −3. Percentages of similarity and identity to each mouse and human protein are listed. (D) The organization of the NMN adenylyltransferase activity center. The key amino acids mutated to reduce enzymatic activity are marked by asterisks (*). The amino acid positions of the nonsense mutations in both alleles are marked by red arrowheads. (E) The *Drosophila* NMNAT protein has similar enzymatic activity as human NMNAT3. The activity is measured by the continuous coupling assay and listed in units per milligram of recombinant protein. The mutant proteins H30A, W98G, R224A, and WR (*W98G/R224A* double mutant) have 1.4%, 22%, 10.8%, and 0.9% of the activity of wild-type protein, respectively.

To identify the molecular lesions of the two alleles of *3R4,* we mapped the lethality associated with this complementation group using *P* element mapping [30]. Fine mapping with five *P* elements pinpointed a 50-kb region at cytological location 96B11 (Figure 2A). Sequencing of 25 kb of genomic DNA showed that both alleles have nonsense mutations in gene *CG13645* (Figure 2B). Expression of the cDNA (green) in photoreceptors rescues both the ERG phenotype and the morphology of synaptic terminals (Figure S1, Table S1). Moreover, a genomic transgene containing the *CG13645* locus (Figure 2B; green) fully rescues the lethality, the ERG defects, and the synaptic defects (Figure S1A–S1D). These data show that *CG13645* corresponds to the gene that is mutated in the *3R4* alleles and that its loss or partial loss causes the observed defects.

The nonsense mutations in both alleles map to the C-terminal half of the predicted protein (Figure 2B), and it is therefore possible that truncated proteins are produced that have residual function. To create null mutations of *CG13645,* we imprecisely excised a *P* element (EY4790) [31] inserted 50 nt upstream of the first exon. Two alleles, *Δ4790–1* and *Δ4790–2,* were generated that cause deletions of the first three or four exons (Figure 2B; blue). These alleles fail to complement the lethality associated with the two EMS-induced alleles, have the same lethal phase (first instar larval stage), display the same ERG defects, and exhibit the same photoreceptor synaptic defects in TEM as the EMS alleles (unpublished data). These data indicate that both EMS-induced alleles are functional null alleles.

*CG13645* encodes NMNAT, a nicotinamide mononucleotide adenylyltransferase, which is a conserved, essential enzyme in most organisms. In humans, three isoforms, NMNAT1, −2, and −3, have been cloned, and their enzymatic properties have been analyzed [32–35]. There are also three predicted genes in the mouse, but only *Nmnat1* has been characterized, mostly as part of the chimeric Wld^s^ protein. *Drosophila CG13645* is equally homologous to mouse and human NMNAT1, −2, or −3 with approximately 45% identity over the entire protein (Figure 2C). Since it is the only NMNAT found in the fly genome, we named it *nmnat*. As shown in Figure 2D, the predicted enzyme activity center consists of an N-terminal catalytic domain and two substrate-binding motifs. The sequence conservation between *Drosophila* NMNAT and other NMNATs within the activity center is greater than 95%. To test whether *nmnat* encodes an active NMN adenylyltransferase, we produced recombinant protein and measured the enzyme activity using a continuous coupling assay. The fly protein has similar NMNAT activity as the human NMNAT3 control. When a single amino acid in the catalytic motif (“H30A”), or two amino acids in the substrate binding motifs are mutated (W98G/R224A and “WR”), the enzymatic activity is reduced to approximately 1% or less of the wild-type protein (Figure 2E). These data indicate that *nmnat* encodes an active NMNAT with a similar enzyme activity center composition as its vertebrate homologs.

**Figure 3:**
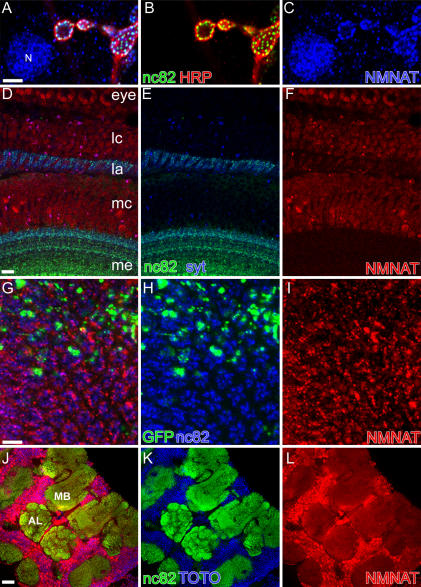
NMNAT Is Highly Enriched in the Nervous System and the Muscle Nuclei (A–C) Third instar larval neuromuscular junction (NMJ) immunolabeled for HRP (red), with nc82 to mark the active zones (green), and for NMNAT (blue). In the NMJ, NMNAT appears as punctae that co-localize with nc82. NMNAT is also enriched in the muscle nucleus (marked by N in [A]). (D–F) Optic lobe (P+60%) immunolabeled for Synaptotagmin (syt) to mark synaptic vesicles (blue), with nc82 to mark active zones (green). and for NMNAT (red). NMNAT is highly enriched in the photoreceptor cell body in the eye and in the cell bodies of different types of neurons in the lamina cortex and medulla cortex, but is present at lower levels in lamina. la, lamina; lc, lamina cortex; mc, medulla cortex; me, medulla. (G–I) MARCM analysis of adult lamina. GFP marks the mutant patches. Laminae are immunolabeled with nc82 to mark photoreceptors (blue) and for NMNAT (red). NMNAT labeling appears as a punctate pattern decorating nc82 labeling, suggesting that NMNAT is present in clusters in photoreceptor terminals. (J–L) Adult brain immunolabeled with TOTO3 to mark nuclei (blue), with nc82 to mark active zones (green), and for NMNAT (red). NMNAT is expressed at higher levels in neuronal nuclei (marked by TOTO3) in the brain and lower levels in the neuropil (marked by nc82). AL, antenna lobe; MB, mushroom body. Scale bars indicate 5 μm.

Both mouse and human NMNAT1 have been shown to primarily localize to nuclei [17,32], whereas human NMNAT3 is present in the cytoplasm and mitochondria [35]. To characterize the localization of the NMNAT protein, we generated a polyclonal antibody against the full-length protein. The specificity of the antibody was verified in mosaic clones of *Δ4790–1* or *Δ4790–2* in eye discs (Figure S2). Mutant patches of *nmnat^1^* or *nmnat^2^* also lack antibody staining at a similar level as mutant patches of *Δ4790–1* or *Δ4790–2* (Figure S2), suggesting nonsense-mediated decay of the transcripts in both EMS alleles, further supporting the genetic data, which indicate that both EMS alleles are null alleles or severe hypomorphs. The antibody enabled us to examine the protein expression pattern by immunohistochemistry in wild-type animals (Figure 3). In third instar larvae, NMNAT is abundantly expressed in neuronal nuclei in the brain and ventral nerve cord (unpublished data) and in muscle cell nuclei (Figure 3A–3C). Relatively low levels of protein are present at the neuromuscular junction where the protein partially co-localizes with the active zone marker monoclonal antibody (mAb) nc82 (Figure 3A–3C). In the pupal optic lobe, NMNAT is expressed in the cell bodies of photoreceptors and numerous optic lobe neurons (Figure 3D–3F). In the adult optic lobe, NMNAT is expressed in the cell bodies of photoreceptors as well as in the terminals of the lamina neuropil. At synapses, NMNAT labeling appears to be punctate where it co-localizes with the active zone marker nc82, suggesting that NMNAT is present in the photoreceptor terminals (Figure 3G–3I). In the adult central brain, the expression persists with high levels in neuronal nuclei and, albeit at substantially lower levels, in synaptic neuropils (Figure 3J–3L). In summary, NMNAT is enriched in the nervous system, both in the neuronal nuclei and the nerve terminals, as well as in the muscle cell nuclei. However, expression of NMNAT in the nervous system using the pan-neuronal driver *elav-GAL4,* in contrast to ubiquitous *actin-GAL4*–driven expression, cannot rescue the lethality associated with the loss of NMNAT protein (Table S1), indicating that NMNAT function is not confined to the nervous system.

### Loss of *nmnat* Causes Severe Early-Onset Neuronal Degeneration

**Figure 4:**
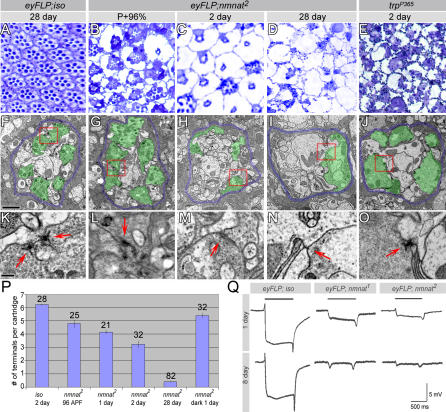
Loss of *nmnat* Causes Severe and Progressive Age-Dependent Degeneration (A–E) Retinal sections of control (iso), *nmnat* mutant eyes of different ages, and a *trp^P365^* mutant eye. Reduced rhabdomeres and vacuoles are seen in P+96%, and both phenotypes become more severe with age. The *nmnat* mutant retina has a more severe phenotype than *trp^P365^* in age-matched animals. (F–J) TEM micrographs of lamina cartridges. Demarcating glia are colored blue and photoreceptor terminals green to accentuate the structures. In the mutant lamina, the number of structurally intact photoreceptor terminals gradually reduces with age. The *trp^P365^* mutant lamina has a rather organized cartridge structure. The red boxes in (F–J) indicate the regions shown in (K–O), respectively. Scale bar in (F) for (F–J) indicates 1 μm. (K–O) Individual synapses boxed in (F–J). In mutant photoreceptors, active zone structures gradually disintegrate with age (arrows); however, the morphology of the T-bars in *trp^P365^* is well preserved. Scale bar in (K) for (K)–(O) indicates 200 nm. (P) Quantification of the number of terminals per cartridge of control (iso) or mutant laminae at different ages. The photoreceptor terminals are recognized by the presence of capitate projections. In mutant laminae, the number of structurally intact photoreceptor terminals decrease with age, and dark rearing can delay the decline. The number of cartridges quantified is indicated above each graph. (Q) ERG recordings of control (iso) and mutant photoreceptors at 1 d and 8 d of age. At 8 d, mutant photoreceptors have minimal responses to light.

The reduced capability of *nmnat* mutant photoreceptors to respond to light, as revealed by ERG, can have several underlying causes: a defect in the phototransduction cascade, or a defect of the structural components of the phototransduction machinery, e.g., in rhabdomeres, the membrane stack in which the phototransduction components reside. We therefore examined the morphology of mutant retinae. The regular organization pattern of ommatidia can be visualized in a retina cross-section (Figure 4A). As shown in Figure 4, in 2-d-old mutant retinae, rhabdomeres are reduced in size, and large vacuoles are abundant in individual ommatidia. This phenotype becomes more severe with age, as in 28-d-old retinae, rhabdomeres are barely recognizable (Figure 4D). Since the exterior morphology of these eyes look normal, we examined the onset of the phenotype and sectioned pupal retinae. At 96 h after puparium formation (APF; P+96%), just prior to eclosion, some defects are already prominent, and the size of rhabdomeres is reduced when compared to those of wild type. However, the phenotype is clearly less severe than in older retinae (Figure 4B versus 4D). TEM of photoreceptor terminals in the lamina (R1–R6 terminals) also reveals defects that follow a similar degenerative profile as that observed at the cell body. At P+96%, synaptic modules, or cartridges, formed by R1–R6 in the lamina are well organized with six photoreceptor terminals per cartridge, and the active zone structures are clearly discernible, indicating that the development of the photoreceptor terminals is mostly normal (Figure 4G and 4L). As the mutant photoreceptors age, the number of recognizable terminals per cartridge declines (quantified in Figure 4P), and the active zone structure becomes smaller and amorphous (Figure 4H, 4I, 4M, and 4N). This phenotype correlates with the progression of the cell body defects. The magnitude of the depolarization and the on/off transients in ERG recordings also decline with age. At 8 d, the ERG response is barely present (Figure 4Q). A comparison with two of the most severe *Drosophila* retinal degeneration mutants, *rdgA (retinal degeneration A)* and *trp^P365^ (transient receptor potential)* [36–39], reveals that *nmnat* mutant retinae have similar but more severe defects (Figure 4C and 4E, and unpublished data). Interestingly, the photoreceptor terminals of both *rdgA* and *trp^P365^* show a rather normal morphology, with correctly organized cartridges containing intact T-bars (Figure 4E, 4J, and 4O, and unpublished data), suggesting that degeneration in these mutants is more restricted to the retina. In summary, in *nmnat* mutant photoreceptors numerous cellular structures are disrupted throughout the entire neuron, from the rhabdomere to the presynaptic terminal, and the phenotype becomes progressively more severe with age.

**Figure 5:**
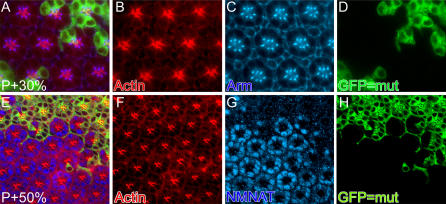
*nmnat* Mutant Photoreceptors Develop Normally MARCM analysis of pupal eye disc at 30 h after puparium formation ( P+30%) (A–D) and 50 h after puparium formation P+50% (E–H). GFP marks the mutant patch in (D) and (H). Anti-Actin antibody labels the rhabdomere structure in (B) and (F). Anti-Armadillo antibody labeling (Arm) marks the adherence junction in (C). Anti-NMNAT antibody shows labeling in wild-type cell bodies, but is dramatically reduced or absent in the mutant patch (G). In both developmental stages, there are no detectable structural differences between wild-type and mutant patches.

To assess whether the development of the photoreceptors and accessory cells are affected in *nmnat* mutant eyes, we compared the morphology of mutant photoreceptors with neighboring wild-type cells by immunofluorescence labeling with several markers. We immunolabeled with antibodies against Actin to reveal rhabdomere structures, Armadillo to reveal the presence and morphology of adherence junctions between photoreceptors, and NMNAT to identify mutant cells. Photoreceptor subtype specification and differentiation are mostly completed by 50% of pupal development [40]. We therefore stained pupal eyes at both 30% and 50% of development and observed no morphological difference between mutant and wild-type tissue (Figure 5), suggesting that mutant photoreceptors develop normally. In addition, TEM at P+96% reveals normal synapse formation by qualitative as well by quantitative criteria (Figure 4G and 4L). We conclude that *nmnat* is not required for photoreceptor differentiation and development, but rather for the maintenance and integrity of mature neurons. In addition, loss of *nmnat* causes a general and severe neurodegenerative phenotype that spans all structures of the neuron including rhabdomeres, cell bodies, axons, and active zones. Hence, *nmnat* is required for neuronal integrity after differentiation.

### Neurodegeneration Caused by Loss of *nmnat* Is Enhanced by Activity

**Figure 6:**
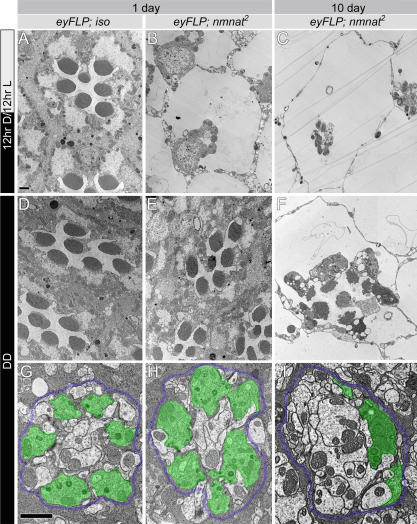
Light Enhances Neurodegeneration in *nmnat* Mutant Photoreceptors (A–C) TEM micrographs of control (iso) or *nmnat* mutant ommatidia at 1 d or 10 d of age kept in 12-hr light/dark cycle (12hr D/12hr L). Note the dramatic reduction of rhabodmere size at 1 d of age (B). This phenotype becomes more severe by day 10 (C). Genotypes and ages are marked on the top of each column. (D–F) TEM micrographs of control or *nmnat* mutant ommatidia at 1 d or 10 d of age reared in constant darkness. Note the dramatic improvement at day 1 ([B] versus [E]) and day 10 ([C] versus [F]). (G–I) TEM micrographs of cartridges containing control and *nmnat* mutant terminals at 1 d or 10 d of age reared in constant darkness. At 1 d of age, the mutant photoreceptor terminals are well organized, compared to the mutant photoreceptors of the same-aged flies raised in regular light/dark cycle (Figure 1H and 1I). Quantification of the number of terminals per cartridge is shown in Figure 4P. Dark rearing does not block synaptic degeneration, as at 10 d of age, the number of structurally intact photoreceptor terminals is reduced (I). Demarcating glia are colored blue and photoreceptor terminals green to emphasize the structures. Scale bars in (A) for (A–F) and in (G) for (G–I) indicate 1 μm.

In an attempt to dissect the mechanisms underlying the neurodegeneration of mutant photoreceptors, we first tested whether the degeneration is light dependent, because a common cause for retinal degeneration is light stimulation [41,42]. Indeed, when flies with *nmnat* mutant photoreceptors are raised in the dark and sectioned at 1 d of age for TEM, they exhibit an overall normal organization of ommatidia similar to wild-type controls (Figure 6D and 6E), a phenotype that is significantly less severe than in flies kept on a 12-h light/dark cycle (Figure 6B). In addition, the morphology of *nmnat* mutant photoreceptor terminals in dark-reared flies is comparable to those of wild-type flies (Figure 6G and 6H), and is significantly less severe than in flies kept on a 12-h light/dark cycle (Figure 1H and 1I). Notably, flies kept in the dark for 10 d exhibit a phenotype that is much less severe than in similarly aged animals kept in a light/dark cycle (Figure 6C). However, signs of degeneration, such as smaller rhabdomeres, the presence of vacuoles in the cell body, and ultrastructural defects in the photoreceptor terminals, are obvious (Figure 6F and 6I). These data show that light dramatically enhances the degenerative process in *nmnat* mutant cells and indicate that the normal function of NMNAT is to provide a potent protection against light-induced degeneration in adults.

**Figure 7:**
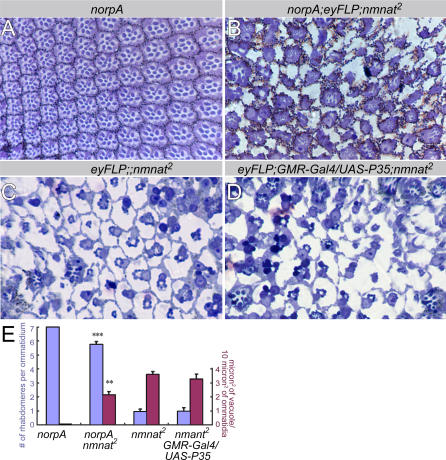
Loss of NMNAT-Induced Degeneration Can Be Attenuated by Blocking Phototransduction and Is Independent of Apoptosis (A) and (B) Ommatidial morphology of 1-d-old *norpA* mutant (A) or *norp; nmnat* double mutant (B) retina. *norpA* mutant retina appears normal at this age. *norpA; nmnat* double mutant retina has greatly improved rhabdomere structure when compared to *nmnat* single mutant (C). (C) and (D) Ommatidial morphology of *nmnat* mutant retina (C) or mutant retina overexpressing P35 (D). There are no detectable differences between (C) and (D), suggesting that P35 overexpression does not rescue the degeneration induced by loss of *nmnat*. (E) Quantification of the number of rhabdomeres and vacuole size for each genotype. Blue columns are the number of rhabdomeres per ommatidium, and the red columns are the vacuole size per 10 μm^2^ of ommatidia. Six animals per genotype and 400 μm^2^ of ommatidia per animal were quantified. Double asterisks (**) indicate *p* < 0.005; and triple asterisks (***) indicate *p* < 0.0005 (Student *t*-test).

To further establish whether neuronal activity is mediating the degenerative process, we tested whether mutants that impair the phototransduction cascade partially protect photoreceptors from neurodegeneration induced by the loss of *nmnat*. *norpA (no receptor potential A)* encodes a phospholipase C, which is required for phototransduction [43]. In *norpA^P24^* mutants, phototransduction is blocked [43–45]. We find that in photoreceptors mutant for both *norpA^P24^* and *nmnat,* neurodegeneration is partially suppressed (Figure 7B and 7C). The overall ommatidial structure is better organized, the vacuoles in the ommatidia are smaller, and the number of rhabdomeres is greater than in *nmnat* mutants (Figure 7E). These observations further indicate that photoreceptor degeneration caused by loss of *nmnat* can be attenuated by reducing neuronal activity.

Next, we tested whether neural degeneration in *nmnat* mutant photoreceptors is related to apoptosis. If so, neuronal degeneration should be suppressed by proteins that inhibit apoptosis. We therefore expressed the apoptosis-inhibiting factor P35 [46] in *nmnat* mutant photoreceptors. We find that P35 expression does not suppress any phenotype associated with the loss of *nmnat* (Figures 7C–7E), suggesting that *nmnat*-dependent degeneration is independent of the P35-mediated apoptotic pathway.

### Enzymatically Inactive NMNAT Rescues Neurodegeneration

**Figure 8:**
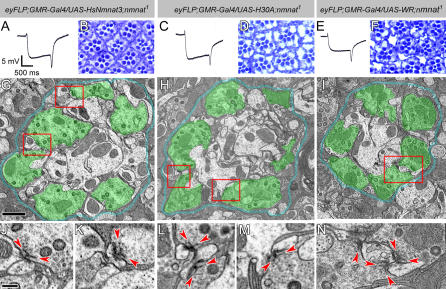
Enzymatically Inactive NMNAT Can Rescue the Neurodegeneration Phenotype Caused by Loss of *nmnat* (A–F) ERG recordings of mutant photoreceptors overexpressing human NMNAT3 (A), or inactive *Drosophila* NMNAT forms H30A (C) or WR (E) in *nmnat* mutant photoreceptors. The genotypes are marked on top of each column. Note that the magnitudes of both depolarization and on/off transients are partially restored. (B), (D), and (F) Retinal structures are partially restored in each genotype. (G–I) TEM micrographs of lamina cartridges. Photoreceptor terminals are well organized in cartridges, similar to wild type. Demarcating glia are colored blue and photoreceptor terminals are colored in green to identify the structures. The red boxes in (G) indicate the regions shown in (J) and (K); the boxes in (H) indicate the regions shown in (L) and (M); and the box in (I) indicates the region shown in (N). Scale bar in (G) for (G–I) indicates 1 μm. (J–N) Individual terminals boxed in (G–I). All active zones have defined platform (arrowheads) and pedestal structures. Scale bar in (J) for (J–N) indicates 200 nm.

In an in vitro culture system, NAD synthesis is required for the Wld^s^ protein to delay axon degeneration [19], and exogenous local application of NAD can prevent axon degeneration [21]. On the other hand, NAD levels were shown to be unchanged in *Wld^s^* axons that show delayed degeneration [12]. If the protective effect of Wld^s^ and NMNAT occurs through enzymatic activity and hence NAD production, enzymatically inactive NMNAT should not rescue degeneration. To test this hypothesis, we generated transgenic flies that encode two different versions of enzymatically inactive NMNAT, *nmnat-H30A,* in which the catalytic center is mutated, and *nmnat-WR,* in which two key residues required for substrate binding are mutated. Both proteins have approximately 1% or less enzymatic activity of the wild-type protein in vitro (Figure 2D and 2E). To our surprise, when the inactive enzymes are expressed in *nmnat* mutant photoreceptors at similar levels as wild-type protein (Figure S3)**,** synaptic structures are fully rescued and rhabdomere size are rescued to 80%–85% of wild type (Figure 8 and Table S1). Importantly, the magnitude of the ERG depolarization in these rescued photoreceptors is more than 75% of wild type, comparable to the rescue with human *NMNAT3* gene *(HsNmnat3)* (Figure 8 and Table S1), indicating that the inactive enzyme can rescue the neuronal degeneration caused by *nmnat* loss of function.

Interestingly, when we express wild-type NMNAT or enzymatically inactive NMNAT in the entire mutant animal using a ubiquitous driver *(actin-GAL4)*, only the wild-type NMNAT is able to rescue the mutant animal to adulthood (Table S1). These data strongly suggest that the mutant enzymes lack activity in vivo and in vitro, and that NMNAT is a multifunctional protein that has two independent roles: NAD synthesis, which is required for viability, and a NAD-independent function that protects against neuronal activity-induced degeneration in mature photoreceptors.

### Overexpression of *nmnat* Protects against Excessive Activity-Induced Neurodegeneration

**Figure 9:**
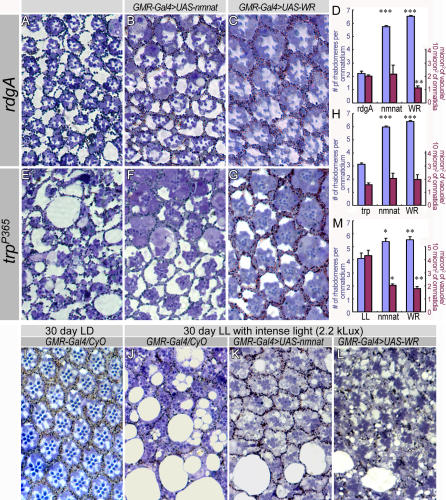
Overexpression of Enzymatically Active or Inactive NMNAT Can Rescue the Neurodegeneration Phenotype Caused by *rdgA, trp^P365^*, and Constant Light Exposure (A–D) Ommatidial morphology of *rdgA* mutant retinae (A) expressing wild-type NMNAT (B) or enzymatically inactive NMNAT (C) at 2 d of age. The quantification of the number of rhabdomeres per ommatidium (blue columns) and the size of vacuoles per surface area (red columns) are displayed (D). The number of rhabdomeres per ommatidium is significantly rescued by expression of wild-type or enzymatically inactive NMNAT. The vacuole size is significantly reduced with the expression of inactive NMNAT but not wild-type NMNAT. (E–H) Ommatidial morphology of *trp^P365^* mutant retinae (E) expressing wild-type NMNAT (F) or enzymatically inactive NMNAT (G) at 2 d of age. The quantification of the number of rhabdomeres per ommatidium (blue columns) and the size of vacuoles per surface area (red columns) are displayed in (H). The number of rhabdomeres per ommatidium is significantly rescued by expressing enzymatically active or inactive NMNAT, although the vacuole size remains unchanged. (I–M) Ommatidial morphology of 30-d-old wild-type flies either kept under ambient light in a 12-h light/dark cycle (I), or under constant intense light (2.2 kLux) (J–L). Overexpression of wild-type NMNAT (K) and the inactive protein NMNAT-WR (L) significantly protect ommatidial morphology compared to wild type (J). Quantification (M) shows an increased number of rhabdomeres per ommatidium (blue columns) and a reduction in vacuole size (red columns). Five animals per genotype and 400 μm^2^ of ommatidia per animal were quantified. A single asterisk (*) indicates *p* < 0.05; double asterisks (**) indicate *p* < 0.005; and triple asterisks (***) indicate *p* < 0.0005 (Student *t*-test).

Delayed Wallerian degeneration in *Wld^s^* mice demonstrates the protective effect of the chimeric gene *Ube4b/Nmnat* [12]. To investigate if overexpression of Drosophila nmnat has protective effects, we tested enzymatically active and inactive forms in hyperactivity-induced neurodegeneration. First, we overexpressed *nmnat* in the retinal degeneration mutants, *rdgA* and *trp^P365^* [36,39,47]. It has been shown that in both *rdgA* and *trp^P365^* mutant photoreceptors, retinal degeneration is caused by constitutive activation of Trp channels [38,39,48,49]. The primary phenotypes of both mutants are loss of rhabdomeres, disorganization of ommatidia, and vacuolarization throughout the retina and photoreceptors (Figures 9A and 9E). Overexpression of wild-type *nmnat* or mutant *nmnat-WR* transgenes partially restores the ommatidial organization (Figure 9A versus 9B and 9C; Figure 9E versus 9F and 9G), significantly reduces vacuole size in *rdgA* (Figure 9D), and causes the presence of a higher number of rhabdomeres per ommatidium than in controls that do not overexpress *nmnat* (Figure 9D and 9H).

In a second paradigm, we induced photoreceptor degeneration by exposing wild-type flies to constant intense light, a strong environmental insult that induces neurodegeneration [50–52]. Thirty days of constant, intense light exposure induces numerous small, as well as very large, vacuoles in wild-type retinae, and a reduced number of rhabdomeres (Figure 9J). In contrast, retinae overexpressing wild-type *nmnat* or *nmnat*-*WR* have significantly reduced size of vacuoles and a higher number of rhabdomeres per ommatidium (Figure 9K, 9L, and 9M). These data show that overexpression of *nmnat,* either wild type or enzymatically inactive, can protect against severe retinal degeneration caused by excessive neuronal activity.

## Discussion

Here we report the identification and characterization of Drosophila nmnat and the first mutant analysis of *nmnat* in any multicellular organism. Our data reveal an essential neuronal protective function for *nmnat,* which is required for neurons to sustain normal neuronal activity. This function is independent of its enzymatic activity and can be exploited to protect neurons against activity-induced neurodegeneration.

### Maintaining Neuronal Integrity

Our mutant analyses provide the first evidence that neuroprotection is a normal function of the endogenous protein, in contrast to previous overexpression reports [16,18]. Several lines of evidence support a role of NMNAT in protecting adult neurons against activity-induced degeneration. First, in the absence of *nmnat,* darkness delays the degeneration process of photoreceptors significantly. Second, mutations that impair components of the phototransduction cascade partially suppress the degeneration caused by loss of *nmnat*. Third, mutants in which photoreceptors degenerate due to constitutive phototransduction *(rdgA* and *trp^P365^)* [38,39,48], are partially protected by overexpression of *nmnat*. Finally, overexpression of *nmnat* in flies that are exposed to intense light potently protects against neuronal degeneration. These observations provide evidence that NMNAT functions to maintain the integrity of mature neurons by protecting them from use-dependent degeneration. This protection is likely to be independent of the NAD synthesis activity of NMNAT, because enzymatically inactive NMNAT proteins protect as effectively as the wild-type protein. Hence, our data indicate that a normal neuronal function of *nmnat* is to protect from activity-induced neurodegeneration. It is possible that the endogenous level of NMNAT is only sufficient to cope with the deterioration caused by normal levels of neuronal activity, but not enough for injury, which would require higher levels of restorative NMNAT. We propose that in the absence of NMNAT, the deterioration caused by normal activity cannot be overcome and is enough to induce degeneration.

### Is NAD Required for the Protective Effect?

The normal development of *nmnat* mutant eyes suggests that *nmnat* is not required for neuronal specification, differentiation, axon pathfinding, or synapse formation. Given that NAD is required for cell survival, it is likely that NAD production by other enzymes compensates for the NAD synthesis function of *nmnat,* because NMNAT catalyzes one of the salvage pathways of NAD synthesis [53]. The de novo and alternative synthesis pathways are catalyzed by enzymes including NAase (nicotinamidase), NaPRTase (nicotinamide phosphoribosyltransferase), and NADS (NAD synthase), and there are predicted genes with each of these enzymatic activities in the fly genome [53] (see Figure S4).

It has been shown in an in vitro culture system that NAD can protect injured axons from degeneration [19,21]. However, this protective effect is not specific to NAD, because pyruvate or EGTA have similar effects in the same studies [21]. It is equally possible that exogenously applied NAD can “free up” NMNAT from its NAD synthesis function and allow more NMNAT to engage in the protective function, thereby delaying degeneration. More puzzlingly, Araki et al. [19] showed that enzymatically inactive NMNAT bearing a mutant substrate binding motif (W170A) cannot delay axon degeneration. Mutation of the equivalent substrate binding motif of the *Drosophila* homolog in our assay reduced its activity to 10.8% of wild type (Figure 2E; W98G). Furthermore, mutations of both substrate binding motifs (Figure 2E; WR) reduce the activity to 0.9% of wild type, which is less than 1/10 that of the single motif mutant, but when expressed in the fly, both mutant enzymes provide an equal level of protection against neurodegeneration. Whether this discrepancy is due to differences in expression levels, or differences between in vitro and in vivo systems, remains to be resolved.

If the neuroprotective effect of MNNAT/Wld^s^ is independent from its NAD synthesis activity, what might be the underlying molecular mechanism? Studies of several neurodegenerative diseases suggest that ubiquitin-mediated proteasome degradation and chaperone-mediated protein folding may play an important role in these degenerative processes [3,54–61]. Studies of a *Drosophila* model of Wallerian degeneration also indicate a protective effect by inhibiting ubiquitination [14]. Interestingly, the N-terminal part of the Wld^s^ fusion protein N70-Ube4b is able to bind an important proteasomal chaperone (VCP) in vitro, and relocates it to the nucleus in cultured cells, suggesting a potential role for chaperones [20].

In conclusion, our genetic and functional analyses present evidence that, in addition to its NAD synthesis activity, a neuronal function of *nmnat* is to maintain neuronal integrity under normal conditions, and neuronal activity potentiates the degeneration that occurs when Nmnat is lost. By extension, more NMNAT protects neurons from neuronal activity induced degeneration. This activity, which is enzyme independent, indicates that the protein has two independent functions: NAD synthesis and maintenance of neuronal integrity.

## Materials and Methods

### *Drosophila* strains and conditions of culture.

Flies were reared at room temperature in ambient light under a normal 12-h light/dark cycle. For dark-rearing experiments, flies were kept in complete darkness from the first instar larval stage onwards. For pupal staging experiments, flies were reared at 25 °C (100% pupal development corresponds to 103 h).

### Antibody production.

The full-length cDNA was cloned into the pET28a (Invitrogen, Carlsbad, California, United States) vector for protein expression (gift of S. Wu). The cDNA fragment was cloned into EcoRI and NotI restriction sites using PCR primers that introduced those sites into the cDNA fragment as described for the cDNA rescue construct. Guinea pig antibodies against this domain were raised by Cocalico Biologicals (Reamstown, Pennsylvania, United States) using the purified recombinant protein. The polyclonal antisera were purified using the Protein A IgG Purification kit (Pierce Biotechnology, Rockford, Illinois, United States). Antibody specificity was confirmed by lack of staining in mutant clonal tissue incubated with anti-NMNAT (Figures 4 and S2).

### *Drosophila* strains, mutagenesis, and screen.

*y w;; P{ry^+t7.2^=neoFRT}82B* isogenized flies were used for mutagenesis and as control animals. Mutagenesis, ERGs, and phototaxis assays were performed as described [24]. The *eyFLP* screen of chromosome arm 3R is described in Mehta et al. [22].

### Construction of rescue constructs and transgenic flies.

Our genomic rescue construct consists of the *nmnat* genomic locus flanked by 0.4 kb of upstream genomic sequence and 0.5 kb of downstream sequence. The following primers were used to amplify this sequence from a clone in P1 phage library containing the CG13645 locus: primer1 5′-accgaattcgagcaggagcccgccacac-3′ and primer 2 5′-ataagaatgcggccgcgtggactcttccaagggaagcaagc-3′. Primer 1 contains an EcoRI site and primer 2 contains a NotI site to facilitate cloning. After sequence verification, the genomic fragment was cloned into the vector pP{CaSpeR-4}, and transgenic flies were generated. For cDNA rescue experiments, we cloned the full coding sequence of cDNA clone AT23490 (Drosophila Gene Collection, http://www.fruitfly.org/DGC/index.html) into pP{UAST} and pP{UAST-HA} (gift from B. Tavsanli), and generated several transgenic lines. The following primers were used to introduce EcoRI and NotI sites at the ends of the *nmnat* coding sequence for ease of cloning: 5′–CCGGAATTCATGTCAGCATTCATCGAGGAAAC-3′ and 5′–TTTTCCTTTTGCGGCCGCAGAGTCGCATTCGGTCGGAGCCG-3′.

### Site-directed mutagenesis and generation of recombinant protein.

Inactive enzyme was created by site-directed mutagenesis of full-length *nmnat* cDNA in pET28a vector using QuikChange Site-Directed Mutagenesis Kit (Stratagene, La Jolla, California, United States). The *H30A, W98G,* and *R224A* mutations were made by one round of mutagenesis, and the *WR* double mutation was made by sequential mutagenesis. The recombinant protein was generated, and the protein concentration was measured by Bradford assay (BioRad, Hercules, California, United States). The mutant cDNAs *nmnat-H30A* and *nmnat-WR* were subcloned into pP{UAST}, and transgenic lines were generated.

### NMNAT activity assay.

Activity of NMNAT (synthesis of NAD) was measured in a continuous coupled enzyme assay by monitoring the increase in absorbance at 340 nm caused by the reduction of NAD to NADH as described [62]. Briefly, the reaction was performed at 37 °C in 16 nM semicarbacide-HCl, 0.625% (v/v) ethanol, 30 nM HEPES buffer (pH 7.4), 12.25 nM MgCl_2_, 1.17 mM ATP, 15 U yeast alcohol dehydrogenase (Sigma, St. Louis, Missouri, United States), purified recombinant NMNAT, and was initiated by adding NMN to a final concentration of 0.625 mM. The activity is determined from the linear progression curve using the following formula:

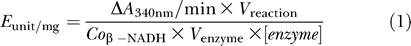
where *Co*_β-NADH_, the extinction coefficient of β-NADH at 340 nm, is 6.22.

### Retina sections and TEM.

Flies of different ages were dissected and fixed at 4 °C in 2% paraformaldehyde; 2% glutaraldehyde; 0.1 M sodium cacodylate; 0.005% CaCl_2_ (pH 7.2), and postfixed in 2% OsO_4_. The 200 nm–thick sections of retina were stained with 1% toludine blue O, 1% sodium tetraborate (Electron Microscopy Sciences, Hatfield, Pennsylvania, United States). The 50-nm thin sections were stained with 4% uranyl acetate and 2.5% lead nitrate. Synaptic features of laminae were scored double-blind by several observers. For photoreceptor terminal quantification, photoreceptor terminals were identified by the presence of capitate projections [27]. In all electron microscopy analyses, we analyzed samples from at least four different animals per genotype.

### Immunocytochemistry.

Eye discs, third instar larval fillets, and larval, pupal, and adult brains were fixed in phosphate buffered saline (PBS) with 3.5% formaldehyde for 15 min and washed in phosphate buffered saline with 0.4% Triton X-100, or with 0.2% Tween 20 for larval neuromuscular junction preparations. Antibody dilutions used: anti-NMNAT 1:1,000; anti-Armadillo 1:200; mAb nc82 1:100; anti-Actin mAb C4 1:200 (ICN Biomedicals, Costa Mesa, California, United States); and TOTO3 1:2,000 (Molecular Probes, Eugene, Oregon, United States). Anti-HRP and secondary antibodies conjugated to Cy3, Cy5, or Alexa 488 (Jackson ImmunoResearch, West Grove, Pennsylvania, United States; and Molecular Probes) were used at 1:250. All antibody incubations were performed at 4 °C overnight in the presence of 5% normal goat serum.

### Image acquisition and processing.

Images from fluorescently labeled specimens were taken on a Zeiss LSM510 confocal microscope (Zeiss, Oberkochen, Germany) and processed using Amira 3.0 (TGS, San Diego, California, United States) and Adobe Photoshop 7.0 (Adobe Systems, San Jose, California, United States).

### Mosaic analyses.

We used the MARCM technique [63] to analyze the localization of various proteins in *nmnat* mutant photoreceptor cells compared to their wild-type neighbors. All eye discs and laminae shown with 50% mutant photoreceptor terminals are from animals of the following genotype: *y w eyFLP GMR-lacZ; P{{w^+^=UAS-mCD8::GFP.L}LL4 / GMR-GAL4; FRT82B nmnat/ FRT82B P{w^+^=tub-GAL80}*. The negatively marked mutant eye disc clones in Figure S2 are from animals of the following genotype: *y w eyFLP GMR-lacZ; FRT82B nmnat/ FRT82B P{w^+mC^=Ubi-GFP.nls}.*

## Supporting Information

Figure S1 Genomic and cDNA Rescue of the Neurodegeneration Phenotypes Caused by Loss of *nmnat*
(A–F) ERG recordings of mutant photoreceptors (A), photoreceptors expressing the CG13645 genomic DNA (C), or photoreceptors expressing CG13645 cDNA driven by *GMR-GAL4* in the *nmnat^1^* mutant background (E). Both genomic DNA and cDNA expression rescue the magnitude of depolarization and on/off transients. The genotypes are marked at the top of each column. (B), (D), and (F) Retinal structures of each genotype. Both genomic DNA and cDNA expression restore the ommatidial morphology.
(G–I) TEM micrographs of lamina cartridges of each genotype. Both genomic DNA and cDNA expression restore the photoreceptor terminal structure and organization. Demarcating glia are colored blue and photoreceptor terminals green. Scale bar in (G) for (G–I) indicates 1 μm.
(J–O) Individual terminals boxed in (G–I). The active zone structures are well organized with defined platform structures (arrowheads), compared to the amorphous structures in the mutant terminals (arrows in J and K). Scale bar in (J) for (J–N) indicates 200 nm.
(8.8 MB TIF)

Figure S2 Polyclonal Antibodies Specifically Recognize NMNAT Protein Mosaic analysis of third instar larval eye disc. Wild-type control *(iso)* or *Δ4790–1, Δ4790–2, nmnat^1^,* or *nmnat^2^* mutant clones are negatively marked. GFP marks the wild-type patch. Eye discs are labeled with NMNAT antibody and TOTO3 to reveal the nuclei. In *Δ4790–1, Δ4790–2, nmnat^1^,* or *nmnat^2^* mutant clones, NMNAT staining is dramatically reduced. The level of reduction in staining in *nmnat^1^* or *nmnat^2^* clones is similar to the level in *Δ4790–1* or *Δ4790–2* clones, suggesting that *nmnat^1^* and *nmnat^2^* are likely protein null alleles. Scale bars indicate 5 μm.
(8.3 MB TIF)

Figure S3 Click here for additional data file. NMNAT and NMNAT-WR Are Expressed at Similar Levels Using the *GMR-GAL4* Driver
Western blot of fly heads showing the level of NMNAT or NMNAT-WR overexpression driven by either *GMR-GAL4* or *elav-GAL4* in a wild-type background. Endogenous NMNAT and overexpressed NMNAT or NMNAT-WR show slightly different migration patterns, in which endogenous protein is highest around 35 kDa (arrow head 1), and overexpressed NMNAT (arrowhead 3) at around 30 kDa and NMNAT-WR (arrowhead 2) at 32 kDa. The difference in size is likely due to post-translational modification. The expression level of NMNAT-WR is slightly lower than NMNAT when driven with *elav-GAL4*. Blotting for Actin was used as a control for equal loading.
(1.3 MB TIF)

Figure S4 NAD Synthesis Pathway NAD can be synthesized through the de novo pathway from L-tryptophan, or two salvage pathways from either nicotinic acid (Na) or nicotinamide (Nam). The fly homologs identified based on sequence homology are shown in red.
(2.6 MB TIF)

Table S1 Rescue Loss of *nmnat* Phenotypes with *nmnat* cDNA Both the wild-type and the inactive enzymes can rescue the morphological and physiological phenotypes of *nmnat* mutant photoreceptors. However, only the wild-type NMNAT, but not the enzymatically inactive NMNAT, can rescue the organismal lethality caused by loss of *nmnat*.
(33 KB DOC)

## Abbreviations

EMS: ethyl methanesulfonate
ERG: electroretinogram
kb: kilobase
NMNAT: nicotinamide mononucleotide adenylyltransferase
1P+96%: 96 h after puparium formation
TEM: transmission electron microscopy
