# Supplementary material for: Drosophila NMNAT Maintains Neural Integrity Independent of Its NAD Synthesis Activity
Source: PLoS Biol. 2006 Nov 28;4(12):e416. doi: 10.1371/journal.pbio.0040416 (PMC1665629; doi:10.1371/journal.pbio.0040416)
Supplement: Supplementary file 1 [file pbio.0040416.st001.doc]

Table S1. Rescue loss of *nmnat* phenotypes with *nmnat* cDNA.

|  |  | driver | *nmnat* | *nmnat-H30A* | *nmnat-WR* | *HsNmnat3* |
| --- | --- | --- | --- | --- | --- | --- |
| Morphology | Retina | *GMR-Gal4* | complete rescue | 85% rescue | 80% rescue | complete rescue |
|  | Synapse | *GMR-Gal4* | complete rescue | complete rescue | complete rescue | complete rescue |
| ERG | depolarization | *GMR-Gal4* | complete rescue | complete rescue | 75% rescue | complete rescue |
|  | on/off transients | *GMR-Gal4* | complete rescue | complete rescue | complete rescue | complete rescue |
| Lethality | | *actin-Gal4* | adulthood | no rescue | no rescue | not tested |
|  |  | *elav-Gal4* | no rescue | not tested | not tested | not tested |

Both the wild type and the inactive enzymes can rescue the morphological and physiological phenotypes of *nmnat* mutant photoreceptors. However, only the wild type NMNAT, but not the enzymatically inactive NMNAT can rescue the organismal lethality caused by loss of *nmnat*.
